# Sharing the View: Obtaining and Sharing Slit-Lamp Microscopy High Quality Images.

**DOI:** 10.51894/001c.164386

**Published:** 2026-08-01

**Authors:** Phillip Whitman, Elizabeth Nisper, Jordan Santz, Matthew K. Hysell

**Affiliations:** 1 Corewell Health Lakeland Hospitals

## 41

### Introduction

In the Emergency Department, Eye complaints are common. Many common diagnoses require use of the Slit Lamp. While thorough documentation helps additional providers moving forward, it does not convey the full picture. Bodies of research in Ophthalmology have attempted to translate Slit-Lamp microscopy images via adapters, cameras, etc. We believe there is a gap in the emergency medicine literature regarding how to adapt these techniques to the emergency department. Therefore, it is important to have a quick, easy-to-follow technique which uses ubiquitous tools (such as smartphones) that most providers will already have on hand. The aim of our current project is to sample different smartphones and test their ability to obtain high-quality images.

### Objectives

Qualitative study comparing common smartphones’ ability to obtain a high-quality image of the eye.

### Methods

Volunteers recruited from amongst medical students, residents, and faculty in the Graduate Medical Education department to test their smartphone’s ability to take photos of the senior author’s eye. Phones divided into overarching categories, Apple and Android. With further division by makes, models, number of cameras, and subjective scoring of image quality.

### Results

Initial qualitative data demonstrated that both Apple and Android phones are useful for obtaining high-quality images. When testing phones, two main themes that emerged, slit-lamp manipulation and number of cameras. Slit-lamp themes included reduction of beam intensity, reducing beam width, and placing beam at an oblique angle to eye improved image quality, reducing flare and preventing overexposure. Cameras were tested with oculars set to 0/0. Single camera phones improved with flash off; both phones with two and three cameras primarily utilized the bottom camera with default settings and automatic enhancements disabled. Within the Electronic Medical Record, outside of flash control, all devices were subject to auto-settings of the phone-based app. While Apple phones were primarily sampled, the few Android devices tested required greater Slit-lamp manipulation and was more sensitive to beam exposure.

### Conclusions

Provided preliminary data that it is possible to document a patient’s slit lamp examination by smartphone without significant manipulation of camera settings or use of third-party adapters.

